# Supplementary material for: Infant Vitamin D Supplements, Fecal Microbiota and Their Metabolites at 3 Months of Age in the CHILD Study Cohort
Source: Biomolecules. 2023 Jan 19;13(2):200. doi: 10.3390/biom13020200 (PMC9952978; doi:10.3390/biom13020200)
Supplement: Supplementary file 1 [file biomolecules-13-00200-s001.zip › biomolecules-2121065-supplementary.pdf]

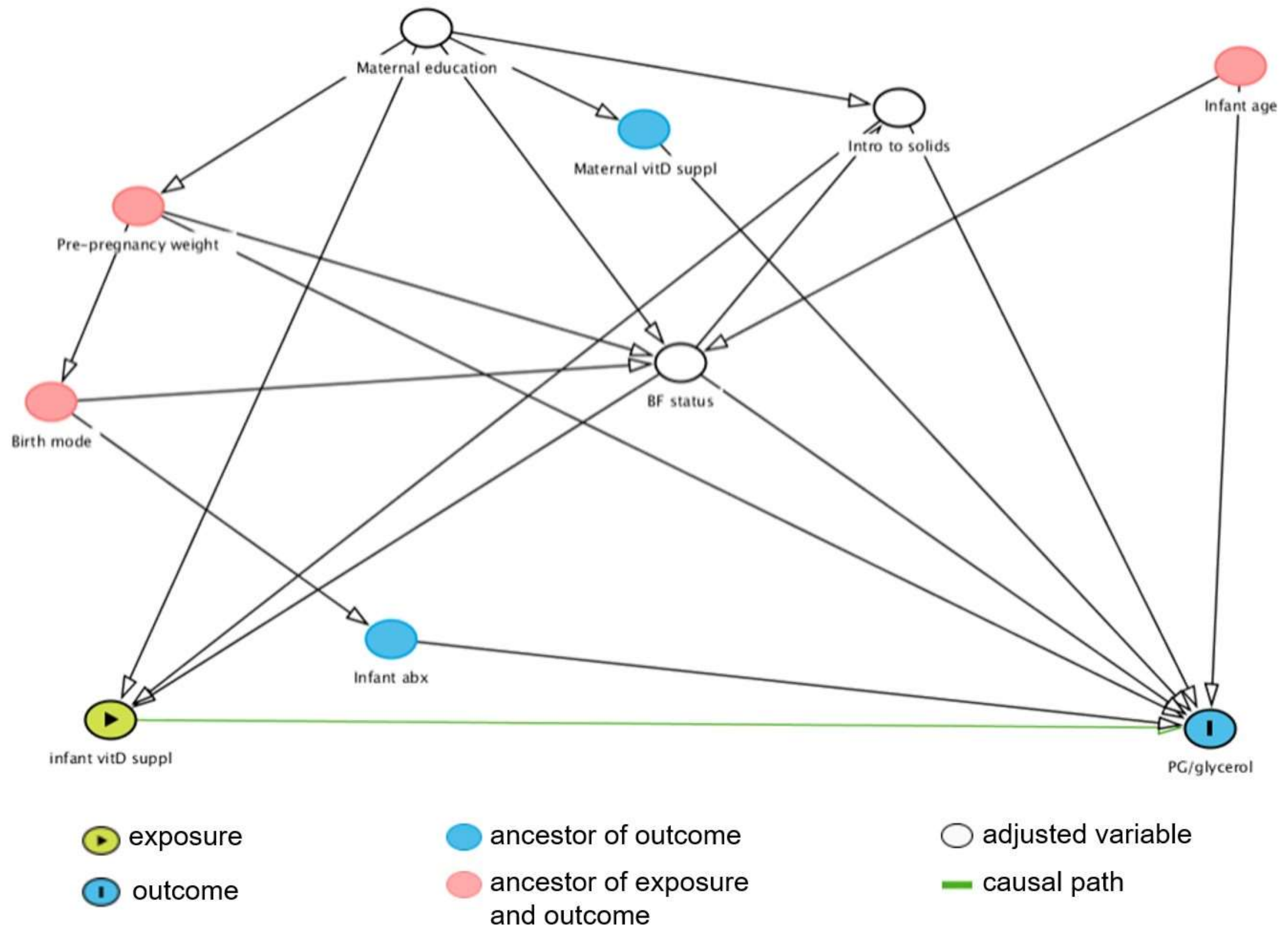
Maternal education
Infant age
Intro to solids
Maternal vitD suppl
Pre-pregnancy weight
BF status
Birth mode
Infant abx
infant vitD suppl
PG/glycerol
exposure
outcome
ancestor of outcome
ancestor of exposure and outcome
adjusted variable
causal path
